# Pre-Symptomatic Detection of Viral Infection in Tobacco Leaves Using PAM Fluorometry

**DOI:** 10.3390/plants10122782

**Published:** 2021-12-16

**Authors:** Alyona Grishina, Oksana Sherstneva, Marina Grinberg, Tatiana Zdobnova, Maria Ageyeva, Andrey Khlopkov, Vladimir Sukhov, Anna Brilkina, Vladimir Vodeneev

**Affiliations:** 1 Department of Biophysics, National Research Lobachevsky State University of Nizhny Novgorod, 23 Gagarin Avenue, 603950 Nizhny Novgorod, Russia; 79159532707@yandex.ru (A.G.); sherstneva-oksana@yandex.ru (O.S.); mag1355@yandex.ru (M.G.); t.zdobnova@mail.ru (T.Z.); anbion@yandex.ru (A.K.); vssuh@mail.ru (V.S.); 2 Department of Biochemistry and Biotechnology, National Research Lobachevsky State University of Nizhny Novgorod, 23 Gagarin Avenue, 603950 Nizhny Novgorod, Russia; ageyevamaria@gmail.com (M.A.); annbril@mail.ru (A.B.)

**Keywords:** biotic stress, chlorophyll fluorescence imaging, pre-symptomatic detection, potato virus X, *Nicotiana benthamiana*

## Abstract

Chlorophyll fluorescence imaging was used to study potato virus X (PVX) infection of *Nicotiana benthamiana*. Infection-induced changes in chlorophyll fluorescence parameters (quantum yield of photosystem II photochemistry (*Φ*_PSII_) and non-photochemical fluorescence quenching (NPQ)) in the non-inoculated leaf were recorded and compared with the spatial distribution of the virus detected by the fluorescence of GFP associated with the virus. We determined infection-related changes at different points of the light-induced chlorophyll fluorescence kinetics and at different days after inoculation. A slight change in the light-adapted steady-state values of *Φ*_PSII_ and NPQ was observed in the infected area of the non-inoculated leaf. In contrast to the steady-state parameters, the dynamics of *Φ*_PSII_ and NPQ caused by the dark–light transition in healthy and infected areas differed significantly starting from the second day after the detection of the virus in a non-inoculated leaf. The coefficients of correlation between chlorophyll fluorescence parameters and virus localization were 0.67 for *Φ*_PSII_ and 0.76 for NPQ. In general, the results demonstrate the possibility of reliable pre-symptomatic detection of the spread of a viral infection using chlorophyll fluorescence imaging.

## 1. Introduction

Phytopathogens are one of the significant causes of the crop losses [1,2]. Despite the fact that currently in agriculture, mainly plant varieties that have a fairly high resistance to infection by various pathogens and their negative impact are used [3,4,5], every year all over the world there is a decrease in crop yields by 10–20% due to plant diseases [6], which represents a serious threat to global food security. Most often, plant diseases are caused by microorganisms of various taxonomic groups, including bacteria, fungi, and viruses. In particular, viruses that infect the Solanaceae are a serious threat, affecting such important agricultural crops as potatoes, tomatoes, peppers, eggplants, and tobacco [7,8,9].

The penetration of viruses into plants occurs with the participation of insects, nematodes, and parasitic fungi, as well as after injury by agricultural tools, from infected plants through grafting, or “vertically” through the seeds or pollen of infected plants [10]. In a plant cell, viruses use the host’s genetic and protein synthesis apparatus for reproduction, and spread throughout the plant using movement proteins (MPs) [11] from cell to cell through plasmodesmata and systemically through phloem sieve elements [12]. The mechanisms of plant defense against viruses include callose deposition in cell walls, membrane structural changes, proteolysis of viral proteins, RNA interference aimed at destroying viral RNA, and transcriptional gene silencing (TGS), as well as a hyper sensitive response (HR) leading to the formation of localized necrotic foci, and the development of systemic acquired resistance (SAR) [12,13]. Despite the presence of protective mechanisms, in most cases, the interaction between viruses and crop plants adversely affects the physiology and morphology of the host plant, causing the development of disease symptoms, leading to a slowdown in plant growth, reduced yield, or death.

Early detection of the initial stage of the disease is especially important, since it al-lows both to reduce crop losses by timely localization of the area of infection, and to im-prove its quality through the use of pesticides and fungicides in a smaller amount. In current agricultural practice, the presence of diseases in plants is most often assessed visually; however, this approach cannot be considered sufficiently reliable and objective. Along with traditional visual diagnostics, ELISA, PCR, sequencing, and fluorescence in situ hybridization (FISH) methods are used to accurately determine plant diseases [14,15,16,17,18]. These methods make it possible to precisely detect the pathogen and determine its taxonomic affiliation. They are constantly being improved in order to increase the speed, sensitivity, and accuracy of the analysis [19,20,21,22,23,24,25]. Despite the high sensitivity and specificity, these methods cannot be considered optimum for mass screening due to their laboriousness, low speed, and high cost of analysis. Therefore, for the screening of diseases of plants growing in large areas of indoor and open ground during mass production of agricultural crops, it is required to use methods that combine cost-effectiveness, high reliability of pathogen detection, and the possibility of automation.

Currently, approaches based on the use of optical methods for detecting pathogens, including fluorescence, multispectral and hyperspectral imaging, and thermography, are actively developing [21,26,27,28]. The advantages of optical methods are their non-invasiveness, high sensitivity, and high analysis speed [29]. The use of such methods is possible due to changes in the plant during the pathogen–host interaction. As a rule, infection reduces the intensity of transpiration, which allows the detection of pathogens by increasing the temperature using thermal imagers [7,27,30,31]. In addition, the development of the disease causes significant alterations in photosynthesis due to changes in the structure and activity of the photosynthetic apparatus, in particular, a decrease in chlorophyll content due to the direct effect of viruses on enzymes of its synthesis and chlorophyll-binding proteins, or a decrease in the rate of CO_2_ assimilation and the efficiency of using the energy of absorbed light in the light-dependent stage of photosynthesis [20,32,33,34,35]. Changes in the content of pigments and the structure of chloroplasts determine the informativeness of spectral methods in detecting infection [36,37,38]. One of the most promising optical methods for investigating infection-induced changes in light reactions of photosynthesis is the imaging of pulse-modulated chlorophyll fluorescence (Pulse-Amplitude-Modulation (PAM) fluorometry). The parameters determined by PAM fluorometry reflect the activity of photosynthesis, the efficiency of which changes rapidly under various external influences [20,39,40,41]. Therefore, the PAM-fluorometry method has a high sensitivity to the detection of various abiotic and biotic stresses, since physiological disturbances begin earlier than structural changes. The high sensitivity of the method in combination with an advanced and constantly improving instrumental base makes the PAM fluorometry one of the most popular in the study of the pathogen–host interaction. In particular, the parameters of chlorophyll fluorescence (ChlF) have shown high sensitivity in the detection of pathogens such as bacteria *Dickeya dadantii*, *Pythium irregulare*, *Pseudomonas syringae*, fungus *Rosellinia necatrix*, viruses *Pepper mild mottle virus (PMMoV)*, *Grapevine leafroll associated virus 3 (GLRaV-3)*, and others [7,9,27,28,30,42,43,44,45].

The most widely used ChlF parameters for detecting infection include the maximum quantum efficiency of photosystem II (F_v_/F_m_) reflecting the fraction of functioning photosystems, effective quantum yield of photosystem II (*Φ*_PSII_), which reflects the efficiency of using the energy of the absorbed light, and non-photochemical quenching of fluorescence (NPQ), which is an indicator of the thermal dissipation of the energy of the absorbed light. In most cases, infection causes a decrease in the linear electron flux in the chloroplast electron transport chain, which manifests itself in a decreased values of F_v_/F_m_ [7,22,43,44,46,47,48] and *Φ*_PSII_ [7,8,22,43,44,46,47,48,49], as well as in an increased NPQ values [7,8,22,27,43,44,46,47,50]. A number of studies have shown the opposite changes, including infection-induced increase in *Φ*_PSII_ [9] and decrease in NPQ [44,48]. In [50], a significant increase in NPQ in the infected area was revealed along with the absence of changes in *Φ*_PSII_. Such contradictory data indicate a different diagnostic potential of different ChlF parameters and requires a detailed study of the ways of the infection influence on the photosynthetic activity parameters.

In some studies, the possibility of detecting pathogens before the appearance of visual symptoms of infection using ChlF analysis has been demonstrated [30,43,44]. The high potential of PAM fluorometry in the pre-symptomatic detection of pathogens has prompted studies aimed at developing and optimizing protocols for ChlF parameters recording, which provide high sensitivity and specificity of the method [7,42,44]. In addition, the promise of using fluorescence imaging for detecting infection can be increased by automating the processing of images reflecting the spatial distribution of the pathogen and photosynthetic activity changes [42,51], and their quantitative assessment [27,44].

At the same time, in order to assess the effectiveness and sensitivity of pre-symptomatic diagnostic methods, it is necessary to control the localization of pathogens, distinguishing between infected and uninfected zones, including the case of systemic spread of the pathogen far from the inoculation zone. One of the most effective approaches for accurate in situ detection of pathogen localization is the use of fluorescently labeled pathogens [52,53,54,55]. This approach will make it possible to correctly determine the recorded parameters with the highest diagnostic potential.

The aim of this work was to study the possibility of pre-symptomatic detection of the potato virus X systemic spread based on the registration of ChlF parameters using PAM imaging in *Nicotiana benthamiana*.

## 2. Results

### 2.1. Dynamics of Virus Spread

**Figure 1:**
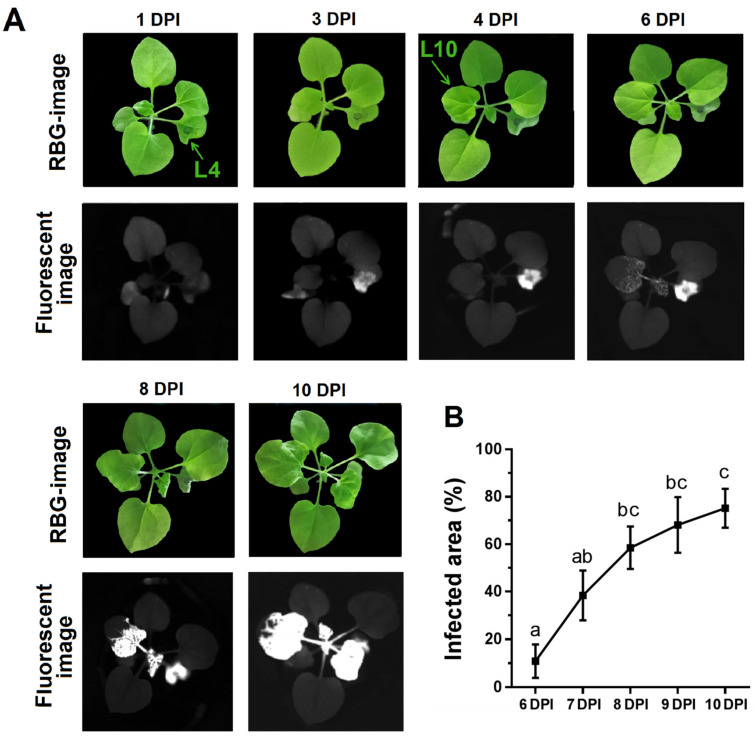
Distribution of PVX-GFP in the *N. benthamiana* plant at various days post-inoculation (DPI): (**A**) RGB and fluorescence (λ_ex_ 452/45, λ_em_ 535/43 nm) images of whole plants obtained using surface fluorescence imaging. (**B**) Dynamics of the PVX-GFP infection area of the tenth tobacco leaf (*n =* 5). Values are mean ± SEM. L4 is the fourth true leaf through which the plants were infected; L10 is the tenth leaf in which the systemic spread of the virus was recorded. Different letters indicate significant differences between treatments at *p* < 0.05 according to Duncan test.

Analysis of fluorescent images of infected tobacco plants, obtained using surface imaging based on the PVX-GFP signal, was carried out to assess the spread of the virus from the zone of inoculation through the plant shoot (Figure 1A). A strong fluorescent signal of PVX-GFP in the inoculated leaf was observed from 3 to 7 days post-inoculation (DPI). This leaf became covered in necrotic lesions at 5–7 DPI and died by the end of the experiment.

Non-inoculated leaves, which had no visible signs of infection, showed PVX-GFP fluorescence at 5-6 DPI, indicating systemic spread of the virus. The fluorescence signal of PVX-GFP was detected in the 10th and 11th leaves at 5–6 DPI; in other leaves, PVX-GFP fluorescence was detected later. In a non-inoculated leaf, the first visual symptoms of PVX infection (mosaic symptom) were detected 2–4 days after the appearance of PVX-GFP fluorescence signal (Figure 1A). 

**Figure 2:**
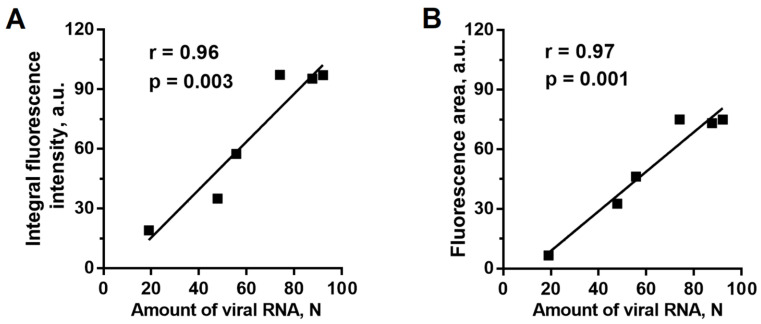
Correlation of the integral fluorescence intensity of PVX-GFP (**A**) and the fluorescence area (**B**) versus the viral RNA amount (*n =* 6). N is an arbitrary unit equal to 2^−Ct^ × 10^4^. The Ct values are shown in Table S1.

The dynamics of the infected area within the analyzed leaf (the 10th leaf in these and subsequent measurements) was assessed by the change in the area from which the PVX-GFP fluorescence was registered (Figure 1B). During the first three days after the detection of the virus in the 10th leaf, the infected area grew rapidly; furthermore, the spread of the virus slowed down. The total infected area was about 80% of the lamina by the end of the experiment (10–12 DPI). The area of the infected zone, assessed by the area of PVX-GFP fluorescence, and the integral fluorescence signal of PVX-GFP showed a strong relationship with the amount of virus RNA in the studied leaves, determined by real-time polymerase chain reaction (rtPCR) (Figure 2); the correlation coefficients were 0.97 and 0.96, respectively. 

### 2.2. Identifying Healthy and Infested Leaf Areas

**Figure 3:**
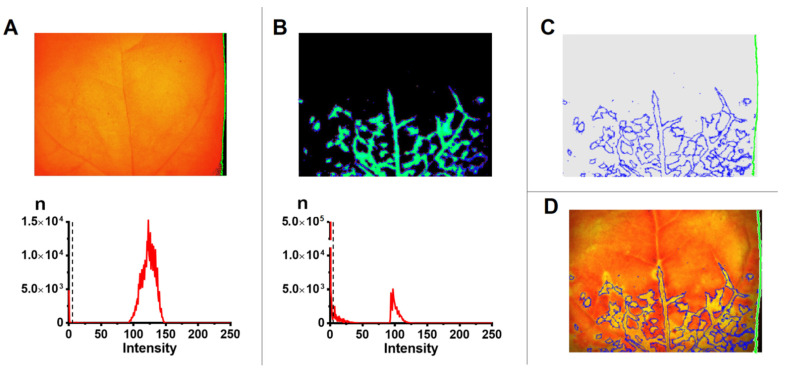
Determination of the infected, border and healthy areas in tobacco leaf images: (**A**) determination of leaf area in the image from chlorophyll fluorescence. The histogram of the signal intensity distribution is shown below. The dashed line in the diagram shows the intensity threshold above which a pixel in the image is defined as a leaf; (**B**) determination of the area infected with PVX-GFP. A fluorescence image of a PVX-GFP infected leaf (λ_ex_ 460 nm, λ_em_ 500–540 nm) is shown above. The histogram of the signal intensity distribution is shown below. The dotted line in the diagram shows the threshold intensity value, above which a pixel in the image was determined as an infected area of the leaf. The histogram of the signal intensity distribution in a non-infected leaf is shown in Figure S1; (**C**) the outlines obtained in images (**A**,**B**); (**D**) distinction of the infected and healthy areas by superimposing of the outlines on the *Φ*_PSII_ image of the same leaf.

For further analysis of the relationship of ChlF parameters with infection, three characteristic areas were determined in the image of the studied leaf (Figure 3): (i) the area in which the fluorescence of GFP associated with the virus (infected area) was recorded, (ii) area 5 pixels wide (0.25 mm) adjacent to the infected (border area), and (iii) leaf area surrounding the infected and border areas (healthy area). For the zoning of the leaf into different areas, an automated image processing procedure was developed; its scheme is shown in Figure 3. The analysis was performed on images sequentially obtained using the IMAGING-PAM MINI Version system from one object. The following images were used: chlorophyll fluorescence, PVX-GFP fluorescence, and *Φ*_PSII_ and NPQ images. Using the ImageJ Fiji software, leaf area was separated from the background based on the intensity of the chlorophyll fluorescence signal (Figure 3A). The next step was to determine the infected leaf area based on the threshold fluorescence intensity of GFP incorporated into the PVX virion (Figure 3B and Figure S1), and to isolate the border area of a fixed width. After which the outlines of the selected areas of an individual leaf were superimposed on the *Φ*_PSII_ and NPQ images of the same leaf (Figure 3C,D).

### 2.3. Analysis of ChlF Parameters in Infected and Healthy Leaf Areas

#### 2.3.1. Light-Induced Dynamics of Φ_PSII_ and NPQ

**Figure 4:**
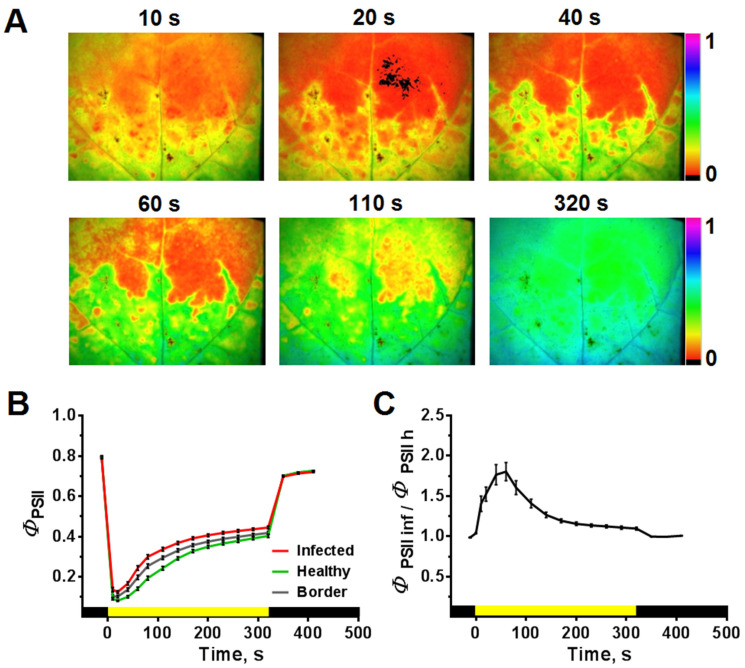
Influence of PVX on the time-course of the quantum yield of PSII (*Φ*_PSII_) (*n =* 25): (**A**) *Φ*_PSII_ images obtained from PVX-GFP infected tenth tobacco leaf at different times after actinic light (AL) was switched on. The color scale bars indicate the *Φ*_PSII_ value given in false colors. (**B**) *Φ*_PSII_ light-induced curves obtained from healthy, infected, and border areas of the leaf. (**C**) The ratios of the *Φ*_PSII_ values of the infected and healthy areas of the leaf. The moment of the AL switching on is taken as zero. Values are mean ± SEM. The ratios of the *Φ*_PSII_ values are statistically significantly differ from 1 in the range from 10 to 320 s (*t*-test, *p* < 0.05). One-way ANOVA was performed to analyze the differences between ChlF parameters ratios (infected/healthy) at different time points of their dynamics (data are shown in Table S2).

**Figure 5:**
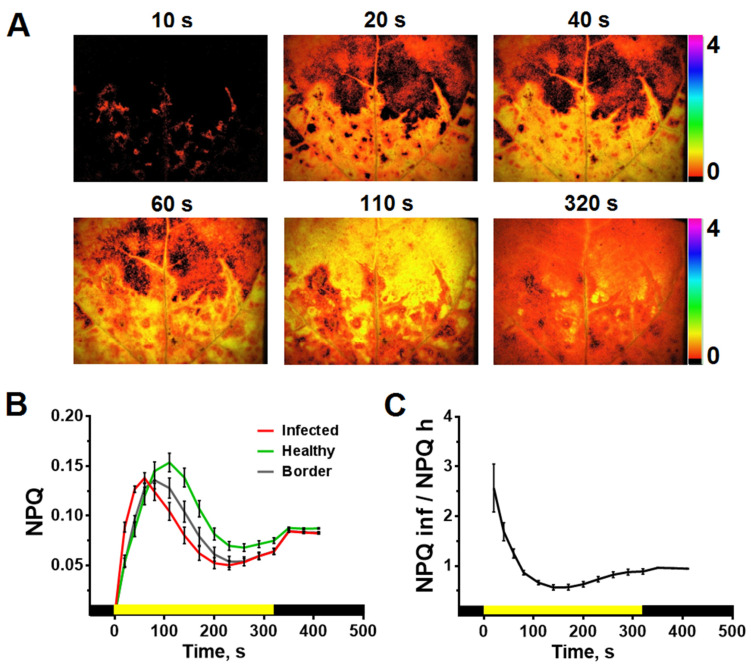
Influence of PVX on the time-course of non-photochemical fluorescence quenching (NPQ) (*n =* 25): (**A**) NPQ images obtained from PVX-GFP infected tenth tobacco leaf at different times after actinic light (AL) was switched on. The color scale bars indicate the NPQ value given in false colors. (**B**) NPQ light-induced curves obtained from healthy, infected, and border areas of the leaf. (**C**) The ratios of the NPQ values of the infected and healthy areas of the leaf. The moment of the AL switching on is taken as zero. Values are mean ± SEM. The ratios of the NPQ values are statistically significantly differ from 1 in the range from 20 to 320 s (*t*-test, *p* < 0.05). One-way ANOVA was performed to analyze the differences between ChlF parameters ratios (infected/healthy) at different time points of their dynamics (data are shown in Table S2).

The dynamics of *Φ*_PSII_ and NPQ induced by actinic light (AL) on/off was recorded in the tenth tobacco leaf of infected plants. Examples of *Φ*_PSII_ and NPQ images obtained at different time intervals after switching on AL are shown in Figure 4A and Figure 5A. Typical light-induced dynamics of *Φ*_PSII_ represents a sharp drop in the *Φ*_PSII_ level to minimum values, followed by recovery and reaching a plateau (Figure 4B). NPQ demonstrates a rapid (tens of seconds) increase to a maximum and its subsequent decrease with reaching a plateau (Figure 5B). The parameters *Φ*_PSII_ and NPQ reach the steady-state level approximately 5 min after the AL was switched on. The light-induced dynamics *Φ*_PSII_ and NPQ differ for the infected and healthy areas of the leaf, the determination of which was performed based on the algorithm described above. Figure 4 and Figure 5 show that the differences are more pronounced for the rates of light-induced transient processes in comparison with the steady-state values of *Φ*_PSII_ (Figure 4) and NPQ (Figure 5). The infected leaf area is characterized by faster light-induced changes in *Φ*_PSII_ and NPQ. The mean time during which *Φ*_PSII_ reaches one-half of its steady-state value (t_1/2_(*Φ*_PSII*320*_)) was 87.8 ± 4.2 and 55 ± 2.6 s, and the mean time of NPQ reaching its maximum (t_(NPQmax)_) was 77.1 ± 4.6 and 34.9 ± 3.1 s for the healthy and infected areas, respectively. The leaf area bordering to the infected zone has an intermediate dynamic of *Φ*_PSII_ and NPQ between the infected and healthy areas.

The times after switching on the AL, for which there are maximum differences between the infected and healthy areas of the leaf, were determined based on the ratio of *Φ*_PSII_ and NPQ in the corresponding areas. The maximum differences occur 40–60 s after switching on the AL for the *Φ*_PSII_ (Figure 4C and Table S2); for NPQ, this time range was 20–40 s (Figure 5C). In further analysis, we used time points of 40 and 60 s after the AL was switched on for NPQ and *Φ*_PSII_, respectively.

#### 2.3.2. The Dependence of *Φ*_PSII_ and NPQ Dynamics on Time after Infection

**Figure 6:**
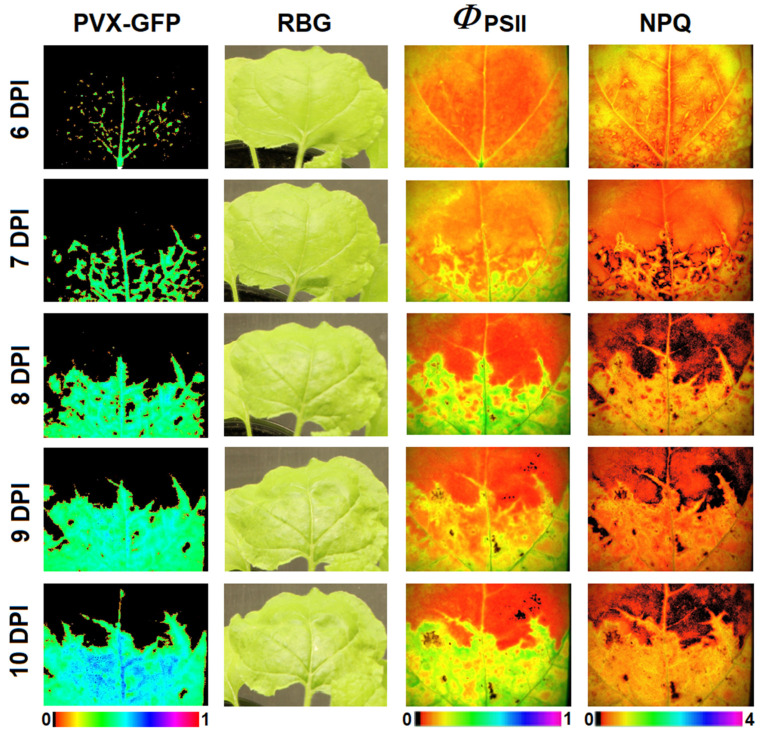
Images of PVX-GFP infected tenth tobacco leaf at various days post-inoculation (DPI): PVX-GFP—fluorescent images (λ_ex_ 460 nm, λ_em_ 500–540 nm); RGB—RGB images; *Φ*_PSII_—*Φ*_PSII_ images taken 60 s after the AL was switched on; NPQ—NPQ images taken 40 s after the AL was switched on; 6 DPI corresponds to the first day of PVX-GFP detection in the studied leaf.

The next step was to analyze the dependence of chlorophyll fluorescence parameters on time after infection. Figure 6 shows the fluorescence images of PVX-GFP in a tobacco leaf and its RGB, *Φ*_PSII_, and NPQ images at 6–10 DPI. The fluorescent signal of PVX-GFP colocalizes with areas of the leaf that have distinct changes in *Φ*_PSII_ and NPQ. The increase in the leaf area affected by the virus is accompanied by the changes in *Φ*_PSII_ and NPQ specific to the infected area. Differences in *Φ*_PSII_ and NPQ between infected and healthy areas were less pronounced in the light-adapted state (Figure S2).

**Figure 7:**
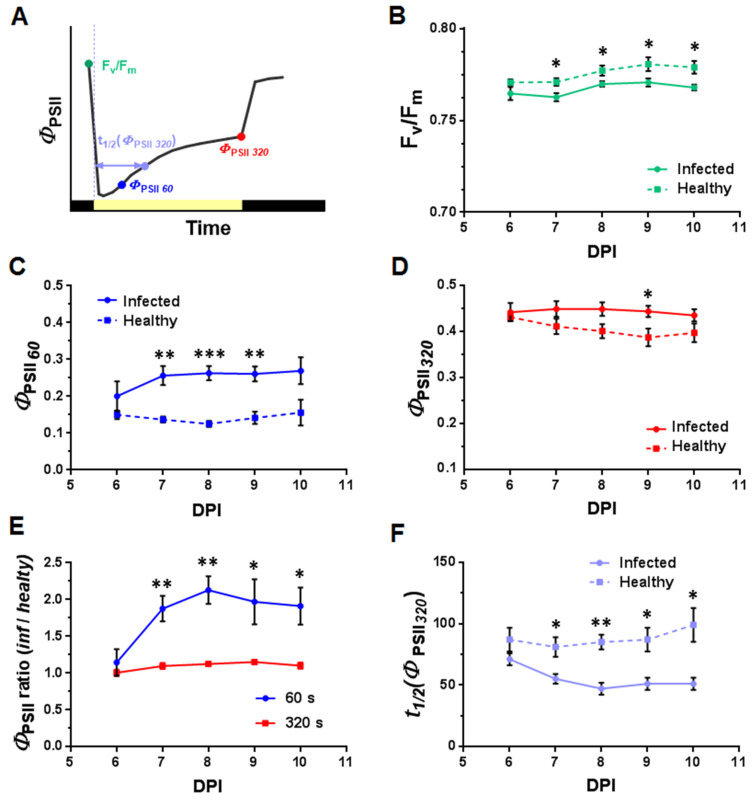
Dynamics of changes in the values of the characteristic parameters of the *Φ*_PSII_ light curve depending on time after inoculation (DPI, day post-inoculation) (*n =* 5): (**A**) A diagram showing the characteristic points of the *Φ*_PSII_ light curve. (**B**) Dynamics of F_v_/F_m_. F_v_/F_m_ was determined for dark adapted leaves after initial exposure of plants to a saturating light pulse. Dynamics of the quantum yield of photosystem II, determined 60 s (*Φ*_PSII*60*_) (**C**) and 320 s (*Φ*_PSII*320*_) (**D**) after the AL was switched on. (**E**) Dynamics of the *Φ*_PSII_ ratio in the infected and healthy areas of the leaf. The ratios are calculated for the *Φ*_PSII_ recorded 60 s (blue curve) and 320 s (red curve) after the AL was switched on. (**F**) Dynamics of the time of reaching 1/2 of the steady-state *Φ*_PSII*320*_ value (t_1/2_(*Φ*_PSII*320*_)); 6 DPI corresponds to the first day of PVX-GFP detection in the studied leaf. Values are mean ± SEM. *, **, *** indicate statistically significant differences between the values in the infected and healthy areas or the differences in the ratios from 1 (*p* < 0.05, 0.01 and 0.001, respectively).

**Figure 8:**
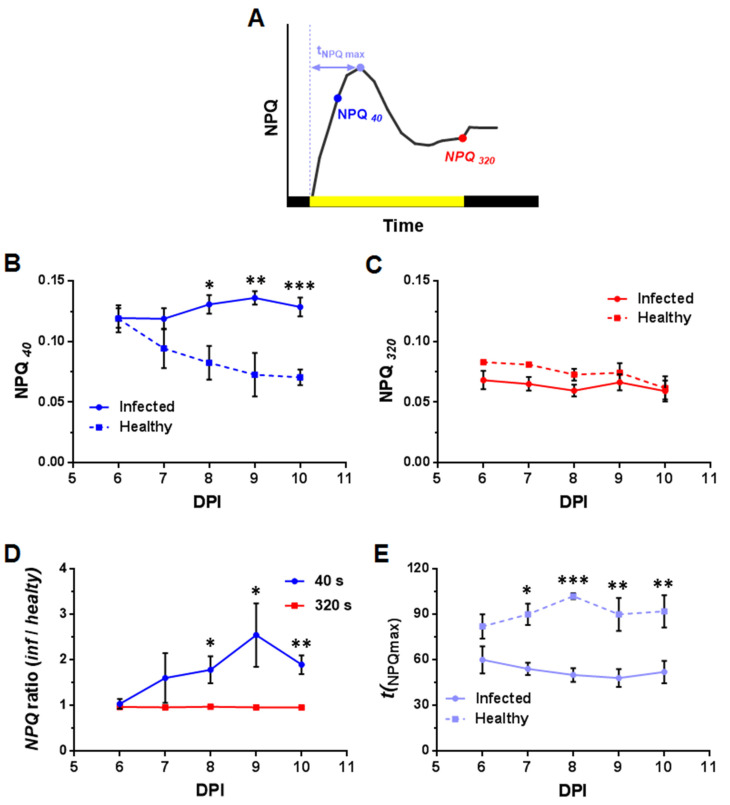
Dynamics of changes in the values of the characteristic parameters of the NPQ light curve depending on the time after inoculation (DPI, day post-inoculation) (*n =* 5): (**A**) A diagram showing the characteristic points of the NPQ light curve. Dynamics of the NPQ determined 40 s (NPQ_40_) (**B**) and after 320 s (NPQ_320_) (**C**) after the AL was switched on. (**D**) Dynamics of the NPQ ratio in the infected and healthy areas of the leaf. The ratios are calculated for NPQ recorded 40 s (blue curve) and 320 s (red curve) after the AL was switched on. (**E**) Dynamics of the time to reach the maximum NPQ value (t _(NPQmax)_); 6 DPI corresponds to the first day of PVX-GFP detection in the studied leaf. Values are mean ± SEM. *, **, *** indicate statistically significant differences between the values in the infected and healthy areas or the differences in the ratios from 1 (*p* < 0.05, 0.01 and 0.001, respectively).

The parameters for which the dynamics depending on time after infection was determined included (Figure 7 and Figure 8; Table S3): the maximum quantum efficiency of photosystem II (F_v_/F_m_), steady-state levels of *Φ*_PSII_ and NPQ in a light-adapted state (320 s after switching on AL, *Φ*_PSII*320*_, and NPQ*_320_*), *Φ*_PSII_ and NPQ values at times for which there is a maximum difference between healthy and infected leaf areas (60 s and 40 s after switching on the AL (*Φ*_PSII*60*_ and NPQ*_40_*, respectively), the time of reaching one-half of the steady-state *Φ*_PSII*320*_ value (t_1/2_(*Φ*_PSII*320*_)), and the time to reach the maximum NPQ value after switching on the AL (t_(NPQmax)_).

F_v_/F_m_, reflecting the fraction of functioning photosystems, has higher values in the healthy leaf area compared to the infected one (Figure 7B). The differences were statistically significant from the second day after the spread of the virus into the analyzed leaf (7 DPI) and remained so during the observation period. In contrast, the effective quantum yield of photosystem II (*Φ*_PSII*320*_) was higher in the infected areas of the leaf (Figure 7C). The differences were more pronounced for the transition state (*Φ*_PSII*60*_) compared to the light-adapted steady state (*Φ*_PSII*320*_) (Figure 7D). The ratio of the values in the infected and healthy areas of the leaf demonstrates that the maximum differences were twofold on the third day after detection of the virus in the leaf (8 DPI) (Figure 7E). t_1/2_(*Φ*_PSII*320*_) decreased with time after infection in the infected areas of the leaf, while in the healthy area t_1/2_(*Φ*_PSII*320*_) shows a tendency to increase (Figure 7F). The differences become statistically significant at the second day of the detecting the virus in the leaf (7 DPI). It should be noted that no differences in ChlF parameters between the 10th leaves of inoculated and control (non-inoculated) plants were observed at 1–5 DPI (before PVX-GFP was detected in the leaf) (Table S4).

The steady-state light-adapted level of NPQ (NPQ*_320_*) in the infected area was slightly lower than in the uninfected area during the entire observation period, but such differences were not statistically significant (Figure 8C). As with *Φ*_PSII_, the differences between the NPQ of healthy and infected areas were more pronounced for the transition state (Figure 8B). Thus, NPQ*_40_* in the infected area of the leaf is statistically significantly higher than that in the healthy area from the third day after the arrival of the virus in the leaf (8 DPI). The time to reach the maximum NPQ after the AL was switched on (t_(NPQmax)_) in the infected part of the leaf was slightly lower from the first day of detection of the virus, and reduced as the time of infection increased (Figure 8E). The difference between t_(NPQmax)_ in the infected and healthy areas became statistically at the second day of PVX-GFP detection in the studied leaf (7 DPI). 

**Figure 9:**
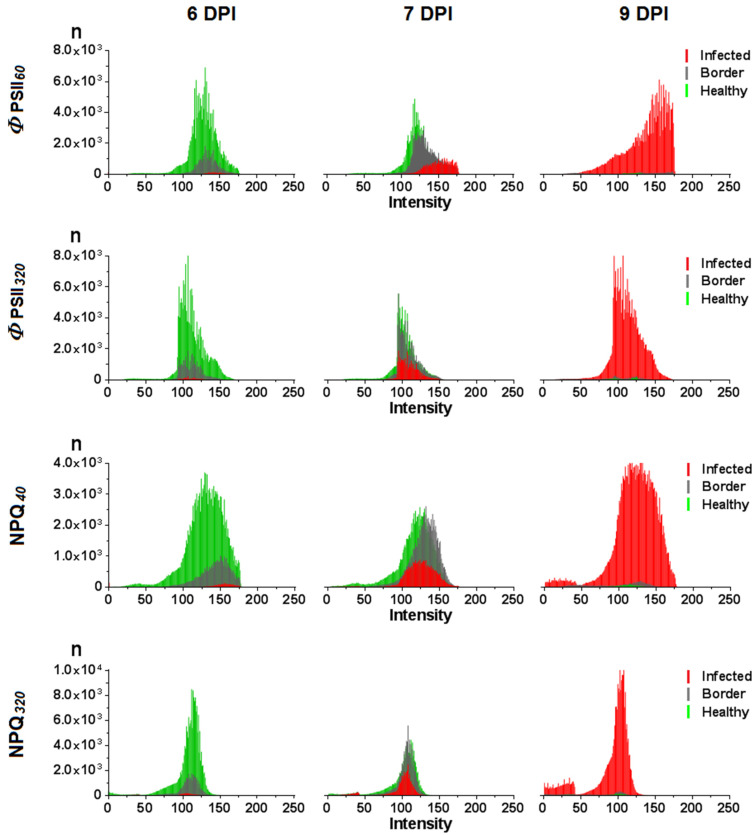
Histograms of the distribution of the intensity of the*Φ*_PSII*60*_ and NPQ*_40_* signal in the infected, border, and healthy areas of the leaf at different days post-inoculation (DPI); 6 DPI corresponds to the first day of PVX-GFP detection in the studied leaf. Histograms of *Φ*_PSII_ images obtained 60 and 320 s after the AL was switched on, and NPQ images obtained 40 and 320 s after the AL was switched on are shown. A representative example of a single plant is presented.

Along with the parameters of the *Φ*_PSII_ and NPQ light-induced curves, determined integrally for the healthy and infected areas of the leaf, the distributions of the signal values were also assessed by individual pixels of the image in the corresponding areas (Figure 9). The study of this distribution is important for the development of a method for automatic image analysis, which is promising for pre-symptomatic diagnostics of plant infection. Distribution analysis was performed on images obtained in time intervals in which there was the maximum difference between infected and healthy leaf areas—*Φ*_PSII*60*_ and NPQ*_40_* (Figure 9. The area of infection was small, and the signal intensity in the infected zone overlapped with the signal intensity in the healthy zone on the first day of virus detection (6 DPI). In most cases there was a distinct difference between the *Φ*_PSII*60*_ and NPQ*_40_* values in the infected and uninfected areas from the second day (7 DPI). The infected zone was characterized by higher values of both parameters. Four to five days after the detection of the virus in the leaf, the area of the healthy zone of the leaf was significantly reduced, the number of corresponding pixels became small, and the signal from the infected area expanded the range of intensities, which led to the overlap of the healthy range by the infected. The distributions of signal intensities in the images obtained in the light-adapted state did not show distinct differences between the infected and healthy leaf areas (Figure 9). The averaged distributions of signal intensities are shown in Figure S3.

### 2.4. Relationship between ChlF Parameters and Infection

**Figure 10:**
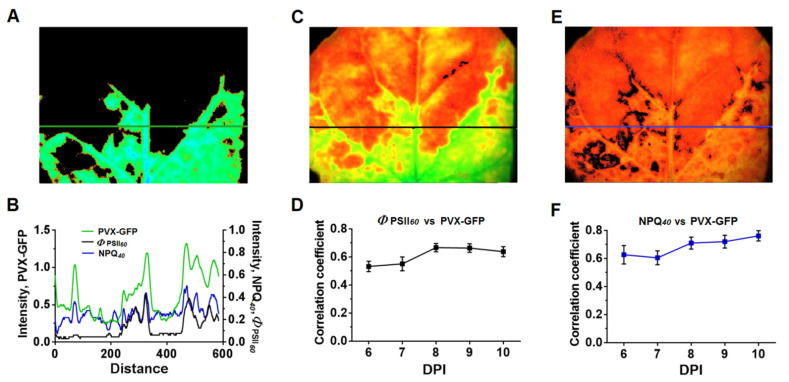
Relationship of ChlF parameters *Φ*_PSII_ and NPQ with infection: (**A**) Fluorescence images of the infected 10th tobacco leaf (λ_ex_ 460 nm, λ_em_ 500–540 nm). (**C**) *Φ*_PSII_ image taken 60 s after the AL was switched on. (**E**) NPQ image taken 40 s after the AL was switched on. (**B**) *Φ*_PSII_, NPQ, and GFP fluorescence profiles determined from the lines shown in (**A**,**C**,**E**). (**D**,**F**) Dynamics of the coefficients of correlation of *Φ*_PSII_ (**D**) and NPQ (**F**) values versus PVX-GFP fluorescence depending on the time after inoculation (DPI, day post-inoculation) (*n =* 5); 6 DPI corresponds to the first day of PVX-GFP detection in the studied leaf. The Pearson correlation coefficients were calculated. Graphs (**D**,**F**) show mean values ± SEM.

A correlation analysis of the relationship between the *Φ*_PSII*60*_ and NPQ*_40_* values and the level of the virus GFP fluorescence signal was carried out to determine the relationship between changes in chlorophyll fluorescence parameters and infection. For this, a leaf profile was drawn through the areas including the infected and uninfected areas using the ImagingWin program, and the intensity of GFP fluorescence, NPQ, and *Φ*_PSII_ was determined along the line (Figure 10). The analysis showed a good agreement of changes in *Φ*_PSII_ and NPQ with the presence of the virus; on the first day of the virus detection in the leaf, the coefficients of correlation of PVX-GFP fluorescence with the *Φ*_PSII_ and NPQ values were 0.53 and 0.62 (*p* < 0.05), respectively). There was a tendency to an increase in the correlation coefficients for both the *Φ*_PSII_ and NPQ values with an increase in the time of infection (Figure 10D,F); the maximum values were 0.67 for *Φ*_PSII_ and 0.76 for NPQ.

Determination of the correlation coefficients for the whole image showed lower values. This could be due to the natural variation in ChlF parameters in the healthy zones of the leaf, while the virus GFP fluorescence intensity was zero.

## 3. Discussion

In our work, we have shown the possibility of detecting the systemic spread of the virus in the host plant by using PAM fluorometry. At the initial stages of infection, PVX-GFP fluorescence was detected in the main veins of the non-inoculated leaf (6 DPI); then the virus spread to the rest of the leaf tissue. This distribution dynamics is consistent with the virus dynamics recorded in other studies, including for PVX [56,57,58]. The use of a virus labeled with the GFP allowed not only to observe the spread, but also to accurately detect the localization of the virus before appearing of visual symptoms of infection in conjunction with the recording of ChlF parameters. This approach made it possible to correctly determine the relationship between changes in ChlF parameters and the presence of a virus in the analyzed part of the plant, as well as to determine the lag period between the appearing of the virus and a change in the recorded parameters. Previously, spatial mapping of the localization of the pathogen, compared with the patterns of ChlF parameters, was carried out only in a few works. In particular, Pérez-Bueno et al. [50] detected the localization of pepper mild mottle tobamovirus (PMMoV-I) in tobacco leaf using specific antiserum against PMMoV-I CP. However, such an analysis performed on detached leaves eliminated the possibility of further assessment of the dynamics of changes

The spread of PVX-GFP causes characteristic changes in chlorophyll fluorescence parameters indicating alterations in photosynthesis. The disturbances caused by viruses include changes in the content of pigments, assimilation of CO_2_, activity of light-dependent reactions, etc. [20,32,34]. Viruses can directly damage the photosynthetic apparatus (modify the structure of membranes, cause clumping of chloroplasts, interfere with their division) [32]; viral proteins can interact with photosynthesis-related proteins, disrupting their function and causing suppression of photosynthetic reactions [32,59]. Virus-induced alterations in photosynthetic activity may be due to changes in gene expression, including genes encoding Rubisco subunits, proteins of the chloroplast electron transport chain, chlorophyll synthesis enzymes, etc. [32,59]. In particular, PVX-induced changes in the level of gene expression, including photosynthesis-related genes, were detected in systemic (non-inoculated) leaves of *N. benthamiana* at 5 DPI [57]. Alterations in synthesis of phytohormones caused by infection can also affect photosynthesis [32,60,61]. Disturbances in the structure and activity of the photosynthetic apparatus cause the observed changes in chlorophyll fluorescence parameters. 

In particular, a decrease in the maximum quantum yield of photosystem II (F_v_/F_m_) was observed starting from the second day after the detection of the virus in the leaf. A decrease in F_v_/F_m_ caused by viruses [46] or other pathogens [7,22,44,47,49] was noted earlier in other works. For example, pea enation mosaic virus caused a decrease in F_v_/F_m_ to 0.65 in non-inoculated leaves of infected pea plants compared to 0.74 in control [46]. A decrease in the maximum quantum efficiency of photosystem II (F_v_/F_m_), which reflects the fraction of functioning photosystems, indicates damage to the photosynthetic apparatus [40,41,62]. Damages caused by pathogens include loss of oxygen-evolving complex proteins, oxidative damage to the thylakoid membrane, etc. [46,50]. It is assumed that impairments in the functioning of the photosynthetic apparatus can be caused, in particular, by reactive oxygen species [20]. The decrease in F_v_/F_m_ recorded in our work does not exceed a few percent, which indicates damage to a small fraction of the photosystems.

Along with a decrease in F_v_/F_m_, we found an oppositely directed change in the effective quantum yield of photosystem II (*Φ*_PSII_), which reflects the ratio of the number of quanta used in photochemical reactions to the total number of absorbed quanta. The light-adapted steady-state level of *Φ*_PSII_ was slightly higher in the infected area in comparison with the healthy one from the second day after the detection of the virus (7 DPI) in the leaf and remained so during the entire observation period. This is inconsistent with the results of a number of other studies, where various pathogens, including the virus, caused a decrease in *Φ*_PSII_ [7,8,22,42,44,46,47,48,49]. It cannot be ruled out that the noted differences in the effect of infection on *Φ*_PSII_ are due to the fact that in most studies the changes were assessed in the inoculated leaf. In addition, Rys et al. [9] showed increased values of *Φ*_PSII_ in an infected leaf in the area surrounding necrotic areas caused by infection. In our experiments, along with an increase in *Φ*_PSII_, a slight decrease in NPQ was observed in the infected area. The light-adapted steady-state level of NPQ in most cases increases under the influence of various pathogens [7,8,22,27,43,44,46,47]. At the same time, in some studies, a decrease in NPQ caused by bacterial [49] and fungal [44] infections was noted, which, in contrast to our work, was accompanied by a decrease in the *Φ*_PSII_ value. It should be noted that the direction of changes in ChlF parameters can be inverted with an increase in the time after infection. In particular, the infection of ginseng plants by root pathogen *Pythium irregulare* caused an increase in the NPQ level by 3 DPI followed by a decrease by 12 DPI [44]. The observed decrease in NPQ is apparently associated with significant damage to the photosynthetic apparatus at the late stages of the development of the disease, which causes suppression of electron transport and regulated quenching processes in photosystem II [44]. Significant damage leading to leaf tissue necrosis was not recorded in our study during the observation period, which explains the absence of such dynamics. In general, PVX-induced changes in *Φ*_PSII_ (Figure 7) and NPQ (Figure 8), recorded in tobacco leaves before the appearance of visible signs of infection, indicate the presence of metabolic disorders in the pre-symptomatic phase of the disease. It is worth noting that our results revealed the difference in both *Φ*_PSII_ and NPQ between the infected and healthy areas, which distinguishes our work from earlier work [50] for the same object (*N. benthamiana*), where the pathogen detection capability was shown for NPQ, but not for *Φ*_PSII_.

In contrast to the steady-state values of the parameters, which in most cases showed small changes in the infected area compared to the healthy one, the light-induced dynamics of ChlF parameters differ significantly. The maximum differences in the values of *Φ*_PSII*60*_ and NPQ*_40_* reached about 50% versus 10–12% for steady-state *Φ*_PSII*320*_ and NPQ*_320_*. Such differences are due to the faster reaction of the parameters to the switching on the actinic light. The time to reach NPQ_max_ (t_(NPQmax)_) and the time to reach one-half of the *Φ*_PSII*320*_ value (t_1/2_(*Φ*_PSII*320*_)) decrease by 45% and 63%, respectively. The acceleration of light-induced NPQ growth has also been shown upon infection of tobacco plants with Potato virus Y [63]. The rate of light-induced changes in *Φ*_PSII_ and NPQ and the time of their reaching the steady-state level are determined by the rate at which equilibrium in production and consumption of ATP and NADPH are reached [49]. Acceleration in reaching steady-state level by photosynthetic activity parameters may indicate a higher rate of ATP consumption in infected cells. This is also confirmed by the higher steady-state level of *Φ*_PSII_ in the infected area. The increased rate of ATP consumption in infected cells is due to the need for additional energy for plant defense processes [64].

It should be noted that the use of the ChlF parameters revealed in our work, recorded in the transition phase of light-induced dynamics, makes it possible to reliably detect the presence of a pathogen several (2–4) days before the first visible signs of infection. Such visual symptoms were mild, and the studied leaf remained viable until the end of the observation. The possibility of pre-symptomatic detection of pathogens using PAM imaging was previously reported in a number of works. In particular, this was shown in relation to viruses [45,49,63,65,66], bacteria [7,28,42,47], and fungi [44,49]. At the same time, in most studies, pathogen detection was carried out in the inoculated leaf. Pre-symptomatic detection of the systemic spread of pathogens in a non-inoculated leaf was demonstrated only for the infection of pea plants with PEMV (pea mosaic virus) [46], as well as the effect of a fungus infecting the roots on ginseng and avocado plants [27,44]. In our work, we compared the dynamics of the spatial distribution of PVX-GFP and ChlF parameters, which made it possible to quantify the relationship between the development of viral infection and changes in photosynthetic activity.

The obtained results show that confident determination of infected leaf areas using ChlF parameters, assessed by PAM fluorometry, requires adherence to the measurement procedure. In particular, a clear distinction into healthy and infected areas on the basis of *Φ*_PSII_ takes place in the range of 40–60 s after switching on the actinic light, and in the range of 20–40 s on the basis of NPQ. This indicates that PAM imaging-based protocols for pre-symptomatic detection of infection should take into account the optimum times to achieve maximum contrast between healthy and infected leaf areas.

## 4. Materials and Methods

### 4.1. Plant Material

The 4–5-week-old *Nicotiana benthamiana* plants were used in the study. Plants were grown in individual 30 mL pots in an environmentally controlled room at 25 °C under 16/8 h (light/dark) photoperiod with cool-white lamp (OSRAM, Munich, Germany; 60 μmol m^−2^ s^−1^).

### 4.2. Agroinfiltration of Plants

We used Potato virus X (PVX), the genome of which contains the GFP gene [55]. During the maturation of viral particles, GFP is incorporated into the capsid, which makes it possible to detect the spread of the virus through the plant using fluorescence imaging methods [48].

The infection of plants of *N. benthamiana* was carried out using the leaf agroinfiltartion. C58C1 strain of *Agrobacterium tumefaciens* with pBin-PVX-GFP or pLH-P19 vectors were used. The vector pBin-PVX-GFP was provided the expression of PVX with GFP (PVX-GFP), and the vector pLH-P19 was provided expression of a protein P19 that acts as a suppressor of RNA interference of plants [12]. The Agrobacterium cultures carrying corresponding binary vectors were kindly provided by Prof. A.G. Solovyev (Lomonosov Moscow State University, A.N. Belozersky Research Institute of Physico-Chemical Biology).

Agrobacteria were grown in Luria Bertani (LB) liquid medium supplemented with rifampicin at 0.1 mg/mL, 10 mM 2-(N-morpholino) ethanesulfonic acid (MES), and 20 µM acetosyringone. Kanamycin (50 μg/mL) or streptomycin (20 μg/mL) and spectinomycin (50 μg/mL) were also added to the culture medium for agrobacterium transformants with the vector pBin-PVX-GFP or vector pLH-P19, respectively.

The agrobacterium transformants with different vectors were cultivated separately at 24 °C overnight using orbital shaker (25 rpm). The overnight agrobacterial cultures (2 mL) of each construct were centrifuged (1000× *g*, 5 min) and cell precipitates were resuspended into 1 mL of an inoculation buffer solution (0.1 M MgCl2, 0.05 M MES, pH 5.5, 150 µM acetosyringone); then the agrobacterial suspensions were left on the orbital shaker for 3 h. After that, three parts of Agrobacterium suspension with the pBin-PVX-GFP vector were mixed with one part of Agrobacteria suspension with the pLH-P19 vector. Inocula were gently infiltrated into the fourth true leaf of *N. benthamiana* plants using syringe. 

### 4.3. Registration of the Spread of a Viral Infection

The spread of PVX-GFP in the plant was assessed using the fluorescence imaging system DVS-03 (ILIT RAS, Russia). GFP fluorescence was excited by the 452/45 nm luminodiode and was emitted by the CMOS-camera (PRIME 95B, Photometrics, Tucson, AZ, USA) with 535/43 nm filter.

The RGB images also were obtained using a Canon EOS 4000D EF-S 18–55 mm reflex camera. Plants were imaged every day from the first day after agroinfiltration for 10 days.

### 4.4. Virus Quantification by Real-Time Polymerase Chain Reaction

The PVX amount in the tenth systemic tobacco leaf was estimated using real-time polymerase chain reaction (rtPCR) at 6–8 DPI. Frozen virus-infected leaves were ground to a fine powder in liquid nitrogen. Total RNA was extracted using ExtractRNA (Evrogen, Russia). Sample quality was examined on a 1.2% agarose gel, RNA concentration was assessed using a NanoVue Plus spectrophotometer (GE Healthcare, Chicago, IL, USA).

The obtained RNA was subjected to reverse transcription reaction using MMLV RT kit with Oligo(dT)17-primer (Evrogen, Moscow, Russia). 

The relative quantification of PVX-GFP was performed with rtPCR using a 7500 Real-Time PCR System and Power SYBR Green PCR Master Mix (Applied Biosystems, USA). Primers (PVX-f: 5′-AAGCCTGAGCACAAATTCGC-3′ and PVX-r: 5′-GCTTCAGACGGTGGCCG-3′) amplifying a 101 bp fragment of the coat protein (CP) gene of PVX were those of [67]. The amplicon size was checked by the separation on a 1.5% agarose gel. The PCR program was as follows: (1) initiation at 50.0 °C for 2 min, (2) “hot start” at 95.0 °C for 10 min, (3) denaturation at 95.0 °C for 15 s, (4) primer annealing and DNA synthesis at 60.0 °C for 1 min. Stages 3–4 were repeated 40 cycles. Determination of Ct (threshold cycle) was performed with 7500 Software v. 2.0.4 (Applied Biosystems, Foster City, CA, USA). The data from the rtPCR experiment were presented as 2^−Ct^ (as described for non-normalized, individual data points in [68]). A content of viral RNA was expressed in arbitrary units as N = 2^−Ct^ × 10^4^.

Prior to virus quantification using rtPCR, fluorescence images of the studied leaves were obtained to determine the area of infection and the fluorescence intensity of PVX-GFP. The procedure for obtaining and processing images is described below.

### 4.5. PAM Imaging

The registration of chlorophyll fluorescence in the infected and uninfected areas of whole tobacco leaves was assessed using PAM fluorometry using the IMAGING-PAM MINI Version (Heinz Walz GmbH, Effeltrich, Germany). The maximum quantum yield of photosystem II (F_v_/F_m_), the quantum yield of photosystem II (*Φ*_PSII_) and non-photochemical quenching of fluorescence (NPQ) were calculated using the Equations [69]:

F_v_/F_m_ = (F_m_ − F_0_)/F_m_, (1)

*Φ*_PSII_ = (F_m_′ − F)/F_m_′, (2)

and

NPQ = (F_m_ − F_m_′)/F_m_′, (3)

where F_m_ is the maximum fluorescence yield of photosystem II, F_0_ is the dark fluorescence yield of photosystem II, and F and F_m_′ are the current and the maximum fluorescence yields of photosystem II under lighting.

The plants were placed in an opaque box containing the IMAGING-PAM MINI Version. The studied leaf was fixed in the holder of the PAM-fluorometer. The size of the image acquisition area was 24 × 32 mm. After the dark adaptation (for 15 min), the dark (F_0_) and maximum (F_m_) yield of fluorescence were measured at the saturation pulse (460 nm, 6 mmol m^−2^ s^−1^, 240 ms duration). Then, the light-induced dynamics of ChlF parameters was recorded, determining the values of the current (F) and maximum (F_m_′) fluorescence yields. Actinic light (AL) (460 nm, intensity 239 μmol m^−2^ s^−1^) was switched on for 320 s; after switching off the AL, the ChlF parameters were recorded for 80 s.

Along with recording chlorophyll fluorescence, the IMAGING-PAM MINI Version system was used to detect PVX-GFP fluorescence from the same leaf area (λ_ex_ 460 nm, λ_em_ 500–540 nm).

### 4.6. Image Processing

Images obtained by the fluorescence imaging system DVS-03 were processed using the open-source software Micro-Manager [70] and ImageJ Fiji [71] Images obtained using the IMAGING-PAM MINI Version were exported using a standard software; further processing of images was carried out using the open-source software ImageJ Fiji. The image was segmented into areas of the leaf and background, infected, healthy (uninfected) and border areas. The leaf and background were determined based on the chlorophyll fluorescence signal; the threshold level was 5 out of 256. The infected area of the leaf was isolated by the fluorescence intensity of PVX-GFP, the threshold level was 5 out of 256.

### 4.7. Statistics

Statistical processing of the results was carried out using MS Excel (Microsoft Corporation, Redmond, WA, USA) and GraphPad Prism software (GraphPad Software Inc., San Diego, CA, USA). Mean values of the studied parameters with standard errors of the mean and the correlation of different parameters (Pearson’s linear coefficient) were calculated. The Kolmogorov–Smirnov test was used for assessing the normality of data distribution. Data were analyzed using one-way analysis of variance (ANOVA) followed by Tukey’s test. Unpaired *t*-test was used to assess the significance of difference between the studied parameters in infected and healthy areas. One-sample *t*-test was used to test if the ratios of ChlF parameters in infected and healthy areas significantly different from 1. The *p*-value was considered significant at *p* < 0.05.

## 5. Conclusions

The presented results demonstrate the potential of using PAM imaging to identify infected and healthy leaf areas. The necessity of preliminary dark adaptation to achieve a high contrast between healthy and infected areas limits the application of the proposed approach in the field. It seems promising to use the proposed method for identifying plants sensitive or resistant to infection in breeding programs due to its high sensitivity in combination with non-invasiveness. Another promising point of application is the screening of the effectiveness of new agrochemicals being developed to control pathogens.

## Data Availability

Data are contained within the article and supplementary material.

## Supplementary Materials

The following are available online at https://www.mdpi.com/article/10.3390/plants10122782/s1, Table S1: Data of real-time polymerase chain reaction; Table S2: The ratios of the ΦPSII and NPQ values in the infected and healthy areas of the leaf, Table S3: Values of the characteristic parameters of the ΦPSII and NPQ light curves depending on time after inoculation; Table S4: Values of the characteristic parameters of the ΦPSII and NPQ light curves in the tenth leaf of inoculated and control (non-inoculated) plants; Figure S1: A fluorescence image and the histogram of the signal intensity distribution in a non-infected leaf, Figure S2: Images of PVX-GFP infected tenth tobacco leaf at various DPI in the light-adapted state; Figure S3: The averaged histograms of the distribution of the intensity of the ΦPSII60 and NPQ40 signal in the infected, border, and healthy areas of the leaf.
