# Supplementary material for: Pre-Symptomatic Detection of Viral Infection in Tobacco Leaves Using PAM Fluorometry
Source: Plants (Basel). 2021 Dec 16;10(12):2782. doi: 10.3390/plants10122782 (PMC8707847; doi:10.3390/plants10122782)
Supplement: Supplementary file 1 [file plants-10-02782-s001.zip › Fig. S1.pdf]

Supplementary Materials

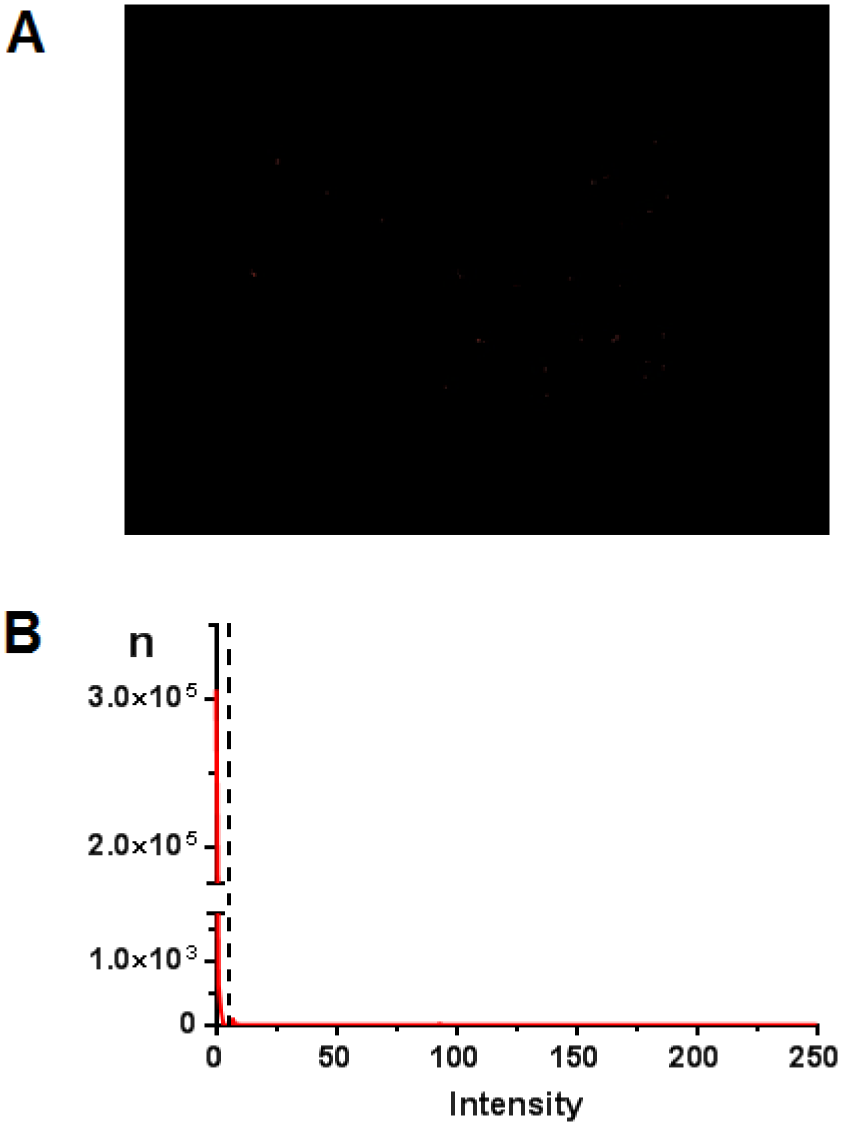

Figure S1. A fluorescence image (λ_{ex} 460 nm, λ_{em} 500-540 nm) (A) and the histogram of the signal intensity distribution (B) in a non-infected leaf. The dotted line in the diagram shows the threshold intensity value, above which a pixel in the image was determined as an infected area of the leaf.
