# Supplementary material for: Pre-Symptomatic Detection of Viral Infection in Tobacco Leaves Using PAM Fluorometry
Source: Plants (Basel). 2021 Dec 16;10(12):2782. doi: 10.3390/plants10122782 (PMC8707847; doi:10.3390/plants10122782)
Supplement: Supplementary file 1 [file plants-10-02782-s001.zip › Fig. S2.pdf]

Supplementary Materials

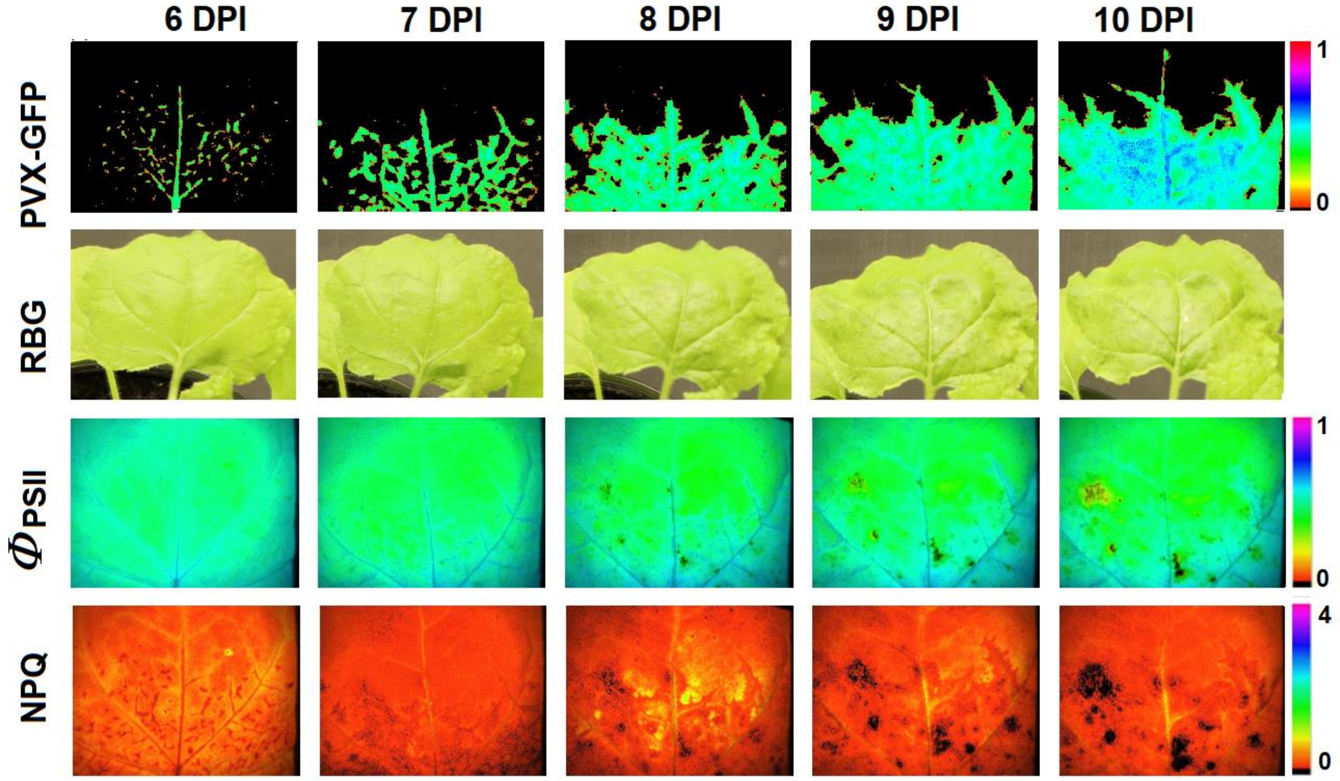

Figure S2. Images of PVX-GFP infected tenth tobacco leaf at various days post-inoculation (DPI) in the light-adapted state. PVX-GFP - fluorescent images (λ_{ex} 460 nm, λ_{em} 500-540 nm). RGB - RGB images. Φ_{PSII} - Φ_{PSII} images taken 320 seconds after the AL was switched on. NPQ - NPQ images taken 320 seconds after the AL was switched on.
