# Supplementary material for: Pre-Symptomatic Detection of Viral Infection in Tobacco Leaves Using PAM Fluorometry
Source: Plants (Basel). 2021 Dec 16;10(12):2782. doi: 10.3390/plants10122782 (PMC8707847; doi:10.3390/plants10122782)
Supplement: Supplementary file 1 [file plants-10-02782-s001.zip › Fig. S3.pdf]

Supplementary Materials

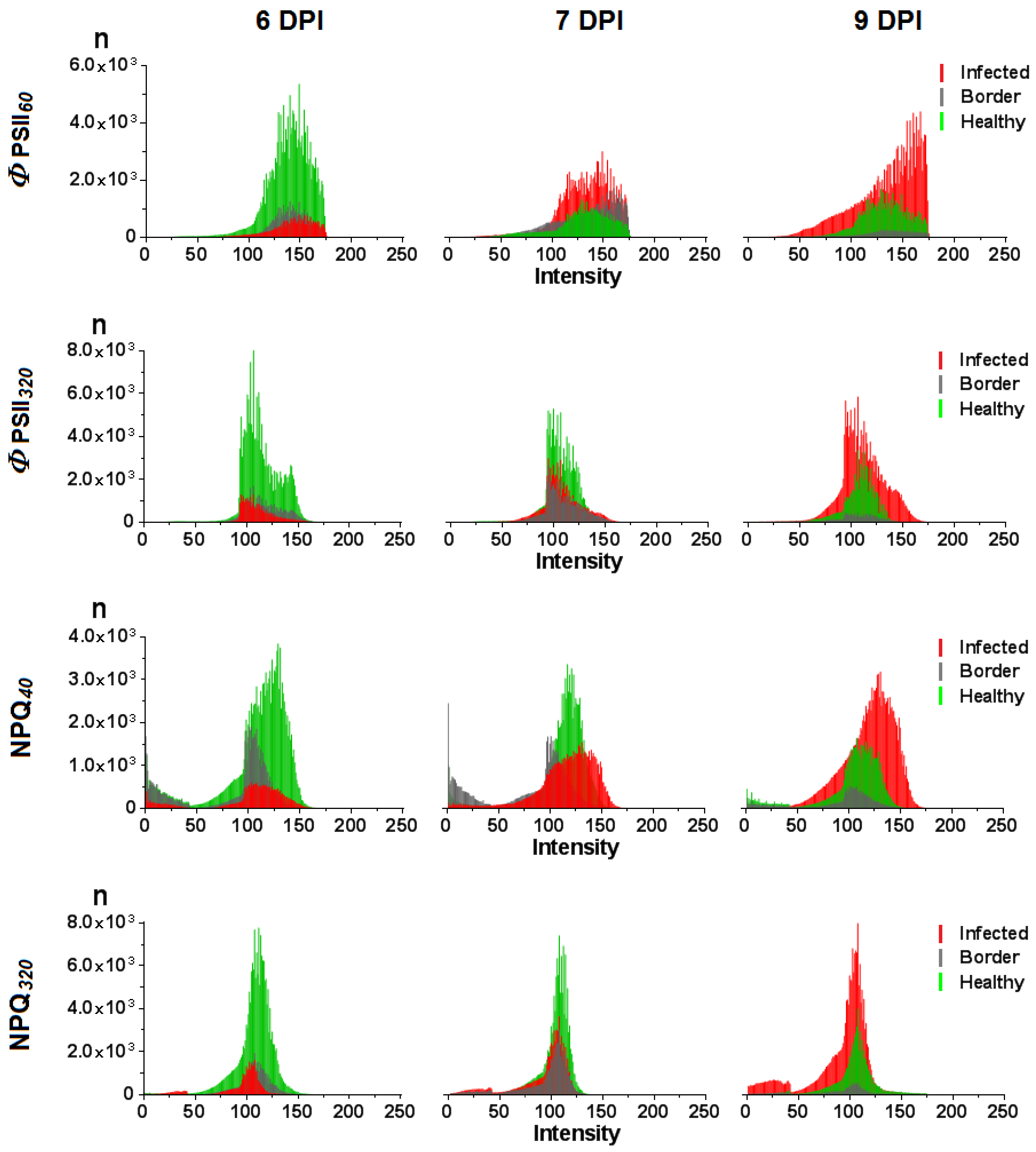

Figure S3. The averaged histograms of the distribution of the intensity of the Φ_{PSII60} and NPQ_{40} signal in the infected, border and healthy areas of the leaf at different days post-inoculation (DPI) ($n = 5$). Histograms of Φ_{PSII} images obtained 60 and 320 s after the AL was switched on, and NPQ images obtained 40 and 320 s after the AL was switched on are shown.
