# Supplementary material for: Pre-Symptomatic Detection of Viral Infection in Tobacco Leaves Using PAM Fluorometry
Source: Plants (Basel). 2021 Dec 16;10(12):2782. doi: 10.3390/plants10122782 (PMC8707847; doi:10.3390/plants10122782)
Supplement: Supplementary file 1 [file plants-10-02782-s001.zip › Table S1.pdf]

Supplementary Materials

Table S1. Data of real-time polymerase chain reaction

Leaf number	Ct_1	Ct_2	Ct_3	N_1	N_2	N_3	Mean N values
**1**	10,48	10,49	10,92	7,00	6,95	5,16	**6,37**
**2**	11,96	12,14	11,93	2,51	2,22	2,56	**2,43**
**3**	9,47	9,47	9,82	14,10	14,10	11,06	**13,09**
**4**	10,66	10,64	10,63	6,18	6,27	6,31	**6,25**
**5**	9,36	9,41	8,93	15,22	14,70	20,50	**16,81**
**6**	9,45	9,48	8,14	14,30	14,00	35,45	**21,25**

* N=2–Ctn×104
