# Supplementary material for: Pre-Symptomatic Detection of Viral Infection in Tobacco Leaves Using PAM Fluorometry
Source: Plants (Basel). 2021 Dec 16;10(12):2782. doi: 10.3390/plants10122782 (PMC8707847; doi:10.3390/plants10122782)
Supplement: Supplementary file 1 [file plants-10-02782-s001.zip › Table S2.pdf]

Supplementary Materials

Table S2. The ratios of the Φ_{PSII} and NPQ values in the infected and healthy areas of the leaf at different time points of the light-induced dynamics *(n = 25)*.

Values are mean ± SEM. The moment of the actinic light (AL) switching on is taken as 0. Different small letters following the data within the same column indicate significant differences (ANOVA followed by Tukey's test, $p < 0.05$). Values with the same letters are not significantly different.

**Time after switching AL on**	**Φ_{PSII} ratio (inf/healthy)**	**NPQ (inf/healthy)**
**−12**	0.986±0.003 ^{a}	
**−11**	0.988±0.005 ^{a}	
**0**	1.039±0.006 ^{a}	
**10**	1.377±0.094 ^{c}	
**20**	1.505±0.086 ^{cd}	2.493±0.467 ^{c}
**40**	1.744±0.122 ^{de}	1.655±0.177 ^{c}
**60**	1.783±0.111 ^{e}	1.236±0.088 ^{bc}
**80**	1.588±0.083 ^{cde}	0.859±0.043 ^{ab}
**110**	1.397±0.052 ^{c}	0.661±0.042 ^{ab}
**140**	1.258±0.032 ^{bc}	0.568±0.05 ^{a}
**170**	1.186±0.024 ^{a}	0.572±0.054 $_{a}$
**200**	1.150±0.021 ^{a}	0.633±0.053 ^{ab}
**230**	1.131±0.019 ^{a}	0.730±0.051 $_{ab}$
**260**	1.118±0.019 ^{a}	0.819±0.057 ^{ab}
**290**	1.103±0.019 ^{a}	0.872±0.056 ^{ab}
**320**	1.091±0.018 ^{a}	0.885±0.051 ^{ab}
**350**	0.995±0.004 ^{a}	0.961±0.006 ^{ab}
**380**	0.994±0.004 ^{a}	0.954±0.006 ^{ab}
**410**	1.006±0.004 ^{a}	0.944±0.006 ^{ab}
