# Supplementary material for: Pre-Symptomatic Detection of Viral Infection in Tobacco Leaves Using PAM Fluorometry
Source: Plants (Basel). 2021 Dec 16;10(12):2782. doi: 10.3390/plants10122782 (PMC8707847; doi:10.3390/plants10122782)
Supplement: Supplementary file 1 [file plants-10-02782-s001.zip › Table S3.pdf]

Supplementary Materials

Table S3. Values of the characteristic parameters of the Φ_{PSII} and NPQ light curves depending on time after inoculation (DPI, day post-inoculation) (*n* = *5*). Values are mean ± SEM.

		6 DPI	7 DPI	8 DPI	9 DPI	10 DPI
F_v/F_m	Infected	0.765±0.004	0.763±0.002	0.770±0.002	0.771±0.002	0.768±0.002
	Healthy	0.771±0.002	0.771±0.002	0.777±0.003	0.781±0.004	0.779±0.003
$\Phi_{PSII320}$	Infected	0.442±0.020	0.449±0.017	0.449±0.015	0.444±0.012	0.435±0.013
	Healthy	0.432±0.009	0.411±0.017	0.401±0.015	0.387±0.019	0.397±0.020
Φ_{PSIId}	Infected	0.699±0.014	0.726±0.003	0.726±0.003	0.732±0.003	0.721±0.005
	Healthy	0.714±0.003	0.722±0.004	0.723±0.005	0.737±0.004	0.730±0.004
$t_{1/2}(\Phi_{PSII320})$	Infected	71±5	55±4	47±5	51±5	51±5
	Healthy	87±10	81±8	85±6	87±10	99±14
NPQ_{max}	Infected	0.177±0.019	0.134±0.005	0.140±0.018	0.140±0.010	0.145±0.012
	Healthy	0.197±0.020	0.160±0.013	0.165±0.022	0.134±0.014	0.146±0.026
NPQ_{320}	Infected	0.068±0.007	0.065±0.006	0.059±0.005	0.066±0.007	0.059±0.009
	Healthy	0.083±0.002	0.081±0.001	0.073±0.005	0.074±0.008	0.062±0.002
NPQ_d	Infected	0.082±0.003	0.082±0.001	0.083±0.001	0.084±0.001	0.086±0.001
	Healthy	0.088±0.002	0.088±0.002	0.086±0.002	0.089±0.002	0.081±0.002
$t_{(NPQmax)}$	Infected	60±9	54±4	50±5	48±6	52±7
	Healthy	82±8	90±7	102±2	90±11	92±11
