# Supplementary material for: Pre-Symptomatic Detection of Viral Infection in Tobacco Leaves Using PAM Fluorometry
Source: Plants (Basel). 2021 Dec 16;10(12):2782. doi: 10.3390/plants10122782 (PMC8707847; doi:10.3390/plants10122782)
Supplement: Supplementary file 1 [file plants-10-02782-s001.zip › Table S4.pdf]

Supplementary Materials

Table S4. Values of the characteristic parameters of the Φ_{PSII} and NPQ light curves in the tenth leaf of inoculated and control (non-inoculated) plants depending on time after inoculation (DPI, day post-inoculation of the fourth leaf) (*n* = *5-8*). Values are mean ± SEM. * indicates the differences between the values in control and inoculated plants ($p < 0.05$).

		1 DPI	2 DPI	3 DPI	4 DPI	5 DPI	6 DPI
F_v/F_m	Inoculated	0.797±0.002*	0.804±0.003	0.805±0.002	0.800±0.003	0.797±0.003	0.793±0.003
	Control	0.787±0.003	0.796±0.002	0.793±0.007	0.800±0.002	0.796±0.001	0.787±0.002
$\Phi_{PSII320}$	Inoculated	0.469±0.016*	0.448±0.019	0.478±0.025	0.440±0.020	0.475±0.020	0.489±0.009*
	Control	0.359±0.018	0.389±0.017	0.409±0.024	0.406±0.048	0.432±0.027	0.421±0.012
Φ_{PSII60}	Inoculated	0.234±0.019*	0.240±0.025	0.290±0.005	0.274±0.040	0.278±0.042	0.299±0.026*
	Control	0.142±0.002	0.189±0.011	0.264±0.012	0.255±0.039	0.218±0.015	0.199±0.007
NPQ_{max}	Inoculated	0.149±0.027	0.150±0.011	0.154±0.013	0.116±0.012	0.098±0.008	0.089±0.008
	Control	0.143±0.005	0.163±0.017	0.142±0.028	0.116±0.013	0.108±0.009	0.100±0.003
NPQ_{320}	Inoculated	0.065±0.004	0.059±0.006	0.061±0.003	0.048±0.004	0.041±0.003	0.040±0.003*
	Control	0.067±0.011	0.055±0.002	0.059±0.005	0.054±0.007	0.051±0.003	0.059±0.003
NPQ_{40}	Inoculated	0.091±0.015	0.074±0.009	0.093±0.015	0.088±0.008	0.080±0.008	0.085±0.013
	Control	0.110±0.017	0.090±0.014	0.112±0.007	0.110±0.005	0.073±0.008	0.088±0.005
